# Apolipoprotein ε in Brain and Retinal Neurodegenerative Diseases

**DOI:** 10.14336/AD.2023.0312-1

**Published:** 2023-08-01

**Authors:** Morteza Abyadeh, Vivek Gupta, Joao A Paulo, Samran Sheriff, Sina Shadfar, Matthew Fitzhenry, Ardeshir Amirkhani, Veer Gupta, Ghasem H Salekdeh, Paul A Haynes, Stuart L Graham, Mehdi Mirzaei

**Affiliations:** ^1^ProGene Technologies Pty Ltd, Sydney, NSW 2113, Australia.; ^2^Department of Clinical Medicine, Faculty of Medicine, Health and Human Sciences, Macquarie Medical School, Macquarie University, Macquarie Park, North Ryde, Sydney, NSW 2109, Australia.; ^3^Department of Cell Biology, Harvard Medical School, Boston, MA 02115, USA.; ^4^Australian Proteome Analysis Facility, Macquarie University, Macquarie Park, NSW 2113, Australia.; ^5^School of Medicine, Deakin University, VIC, Australia.; ^6^School of Natural Sciences, Macquarie University, Macquarie Park, NSW 2109, Australia.

**Keywords:** Apolipoprotein E, Alzheimer’s disease;, age-related macular degeneration, glaucoma, diabetic retinopathy, retinal neurodegenerative disease

## Abstract

Alzheimer’s disease (AD) is the most common form of dementia that remains incurable and has become a major medical, social, and economic challenge worldwide. AD is characterized by pathological hallmarks of senile plaques (SP) and neurofibrillary tangles (NFTs) that damage the brain up to twenty years before a clinical diagnosis is made. Interestingly these pathological features have also been observed in retinal neurodegenerative diseases including age related macular degeneration (ARMD), glaucoma and diabetic retinopathy (DR). An association of AD with these diseases has been suggested in epidemiological studies and several common pathological events and risk factors have been identified between these diseases. The E4 allele of Apolipoprotein E (*APOE*) is a well-established genetic risk factor for late onset AD. The ApoE ε4 allele is also associated with retinal neurodegenerative diseases however in contrast to AD, it is considered protective in AMD, likewise ApoE E2 allele, which is a protective factor for AD, has been implicated as a risk factor for AMD and glaucoma. This review summarizes the evidence on the effects of ApoE in retinal neurodegenerative diseases and discusses the overlapping molecular pathways in AD. The involvement of ApoE in regulating amyloid beta (Aβ) and tau pathology, inflammation, vascular integrity, glucose metabolism and vascular endothelial growth factor (VEGF) signaling is also discussed.

## Introduction

Alzheimer’s disease (AD) is the leading cause of dementia and contributes up to 60-70% of dementia cases worldwide [1, 2]. Currently over 50 million people are living with dementia, with this number predicted to reach 152 million in 2050 [1, 3]. AD is characterized by two well established pathological hallmarks in the form of extracellular senile plaques (SP) and intracellular neurofibrillary tangles (NFTs). Senile plaques are formed from the accumulation of 40 or 42 amino acids amyloid-β (Aβ) peptides derived from amyloid precursor protein (APP), whilst neurofibrillary tangles (NFTs) are composed of hyperphosphorylated tau protein [4-6]. These pathological hallmarks have been the target of major drug developments to modify the disease course however have ultimately not been able to meet the primary end points in majority of clinical trials thereby highlighting the molecular complexity of this disease. It also signifies the importance to identify suitable biomarkers for early detection and management of disease conditions [3, 7-9]. AD is a complex multifactorial disease with a wide range of causal and modifiable risk factors to which aging remains the most prominent and widely studied. Other risk factors that have shown associations with AD include gender, lifestyle, and genetics. In addition, factors such as environmental influences, infectious agents and traumatic head injury have been reported [10, 11]. The Apolipoprotein E (ApoE) E4 allele is the strongest genetic risk factor for the development of AD. The presence of the E4 allele increases one’s genetic risk of developing AD by up to 5-fold, with genotyping and ethnicity increasing this risk to as high as 12 fold [10]. The risk of developing AD is higher in African American/Black and Hispanic/Latino individuals compared with non-Hispanic White individuals, while White individuals showed higher association of E4 with the risk of AD than African American individuals [12, 13]. Also Asssociation of E4 with the increased risk of AD is more tangible in women than men [12]. Patients with AD show symptoms of cognitive impairment such as disorientation, memory loss and socio-behavioral impairments [14]. Vision-related changes are also common in AD patients with a growing number of epidemiological studies indicating a clear association between AD and retinal neurodegenerative disease [15-17]. Amyloid and tau protein aggregates have also been observed in the eyes of patients with retinal neurodegenerative disease including age-related macular degeneration (AMD), glaucoma and diabetic retinopathy (DR) [18-21]. Interestingly ApoE is also a genetic risk factor for developing these retinal neurodegenerative diseases, however, studies have suggested that the E2 allele is a potential risk factor compared to the E3 allele and E4 allele which may be protective [22-24]. This is in contrast to what has been known to occur in AD, where the ApoE2 allele has been suggested to be protective and the ApoE4 as detrimental [9, 25]. This association has recently been explored further to comment on the influence of the APOE as either protective or detrimental on various other diseases. The UK Biobank 2020 study explored the genotype associated risk profile of APOE on over 950 diseases and confirmed a strong association between APOE and both hypercholesteromeria and ischemic heart disease and suggested potential disease protection and detrimental effects of the E2 genotype for several conditions [26].

In this review we summarize recent developments on the involvement of ApoE in AD and retinal neurodegenerative diseases. Here we provide an overview on the suggested mechanisms underlying differential effects of ApoE isoforms in these diseases, with a focus on common pathological events between AD and retinal diseases comprising AMD, glaucoma, and DR.

### Biology of ApoE

Apolipoprotein E is a 299 amino acid amphipathic glycoprotein with a molecular weight of ~ 34 kDa, which plays a key role in lipid and cholesterol transport [27, 28]. The liver is the major site of peripheral plasma APOE production with the majority of lipoprotein subclasses derived from the liver[29]. However it has also been reported that APOE expression has been sustained and observed in non hepatic tissue such as the brain, retina and kidney [30, 31]. The central nervous system is also associated with APOE, it is produced in glial cells, functioning primarily as a transport protein between astrocytes and neurons during growth and repair. As the Blood Brain Barrier (BBB) has limited permeability to intact lipoproteins, pericyets also play a key role in the control of brain blood flow and production of large quantities of APOE [32].

**Figure 1.**
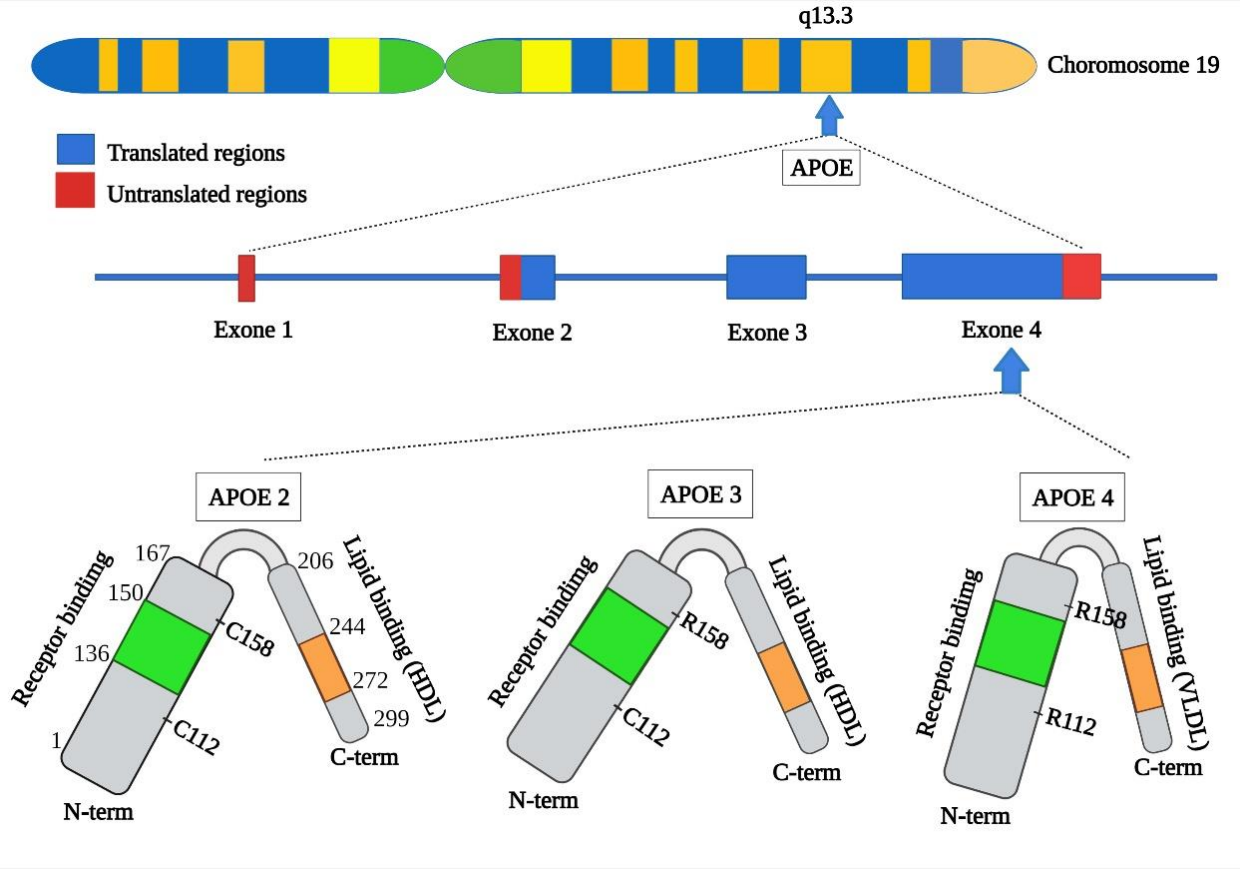
**Schematic illustration of structural and functional regions of ApoE**. *APOE* gene is located on chromosome 19 at position q13.3, polymorphism in this gene results in three different isoforms that have different amino acids at position 112 and 158 including APOE2 (Cys112; Cys158), APOE3 (Cys112; Arg158) and APOE4 (Arg158; Arg158). Apo-E consists of two domains that are joined by a hinge region: the N-terminal domain (residues 1-167) that contains the receptor-binding region (residues 136-150), and the C-terminal domain (residues 206-299) that contains the lipid-binding region (residues 244-272).

The ApoE gene is located on Chromosome 19 and has three variant alleles namely E2, E3 and E4 which generate six genotypes (ε2/ε2, ε2/ε3, ε2/ε4, ε3/ε3, ε3/ε4 and ε4/ε4)[33, 34]. These three isoforms differ only in two amino acids, located at positions 112 and 158 [31]. The molecular composition of the isoforms also varies with the E2 isoform having two cysteines, the E3 isoform having a cysteine and arginine and the E4 isoform having two arginines at these positions respectively (Fig. 1). It should also be recognized that there are also however several lower frequency polymorphisms of APOE, including APOE5 (E5f and E5s) and APOE7 [35]. APOE7 is a rare mutant of the APOE3 conprising two lysine residues replacing glutamic acid at position 244 and 245, that is reported as a risk factor for cognitive impairment, however its association with AD remains unclear [36].

This single amino acid difference has profound effects on protein structure and function. The E3 allele is considered as the parent form with a worldwide frequency of 77.9% , followed by E4 and E2 with frequency of 13.7% and 8.4% respectively [9, 37, 38]ApoE binds to lipoproteins particles (LPPs) such as very low-density lipoprotein (VLDL) and high-density lipoprotein (HDL) and acts as a recognition ligand for low-density lipoprotein (LDL) receptor (LDLR), a receptor family that mediates internalization of lipids [39]. ApoE isoforms have different binding preferences for (LPPs); E2 and E3 bind preferentially to HDL whereas E4 binds to VLDL and E2 binds poorly to the LDLR compared to other isoforms [39]. Consequently, compared to E3, E2 carriers have lower plasma levels of total cholesterol and LDL, and higher levels of HDL and triglycerides (TGs). E4 carriers have higher plasma levels of total cholesterol, TGs and LDL, and lower levels of HDL [9, 37]. ApoE isoforms are only expressed in humans, and other species have only one form of ApoE, which is structurally similar to ApoE4 with two arginines at positions 112 and 158, but functionally similar to ApoE3 [40].

### The role of ApoE in neurodegenerative diseases of the Eye

#### The role of ApoE in the retina

In the retina, ApoE is expressed in various cells including; müller glial, retinal ganglion and the retinal pigment epithelium (RPE) cells [41, 42]. Besides its critical role in lipid metabolism, ApoE has also been reported to participate in extracellular matrix (ECM) remodeling [43]. In diabetic retinopathy particularly, the high level of available glucose increases the production of pro-heparanase by endothelial cells (EC), that are primary cell type which contacts blood and lines all blood vessels and regulates exchanges between the blood and the surrounding tissues[44].Then, this inactive form transfers into the ECM and internalizes through low-density lipoprotein (LDL) receptor-related protein-1 (LRP-1), following internalization, pro-heparanase converts to its active form and degrades heparan sulfate (HS) and triggers downstream events including ECM impairment, EC injury, blood vessel leakage, drusen formation and development of diabetic retinopathy and macular edema [45]. ApoE plays a protective role against ECM impairment through reduced production of active heparanase induced by competitive inhibition of pro-heparanase uptake by LRP-1[46-48]. Moreover, ApoE has been shown to inhibit glutamate-induced apoptosis in retinal ganglion cells (RGCs) through binding to LRP-1[49]. Increased levels of glutamate stimulates NMDA receptors thereby increasing intracellular Ca^2+^ and inducing cytochrome C release from mitochondria promoting RGCs death [49, 50]. Apolipoprotein E-containing lipoproteins (E-LPs) bind to LRP-1 and interact with NMDA receptors and inhibit intracellular Ca^2+^ elevation, therefore play a vital role in protecting RGCs from Ca^2+^ and induced apoptosis [51]. Moreover E-LPs were shown to decrease RGC loss, in a mouse model of normal tension glaucoma in glutamate aspartate transporter-deficient mice [49]. However, there are also conflicting reports indicating that the RGCs in ApoE-deficient mice were significantly resistant to axonal damage-induced retinal ganglion cell death compared to wild type. This interaction suggests a neuroprotective effect for ApoE deficiency against axonal damage-induced RGC death possibly though impaired cytotoxic kainic acid (KA) pathway or stress adaptation [52].

**Figure 2.**
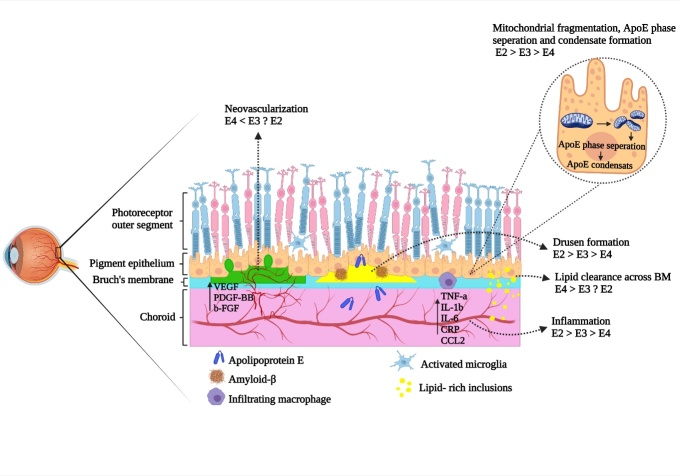
**Schematics of the human eye with age related macular degeneration (AMD) and differential effects of ApoE isoforms on pathological events in AMD**. ApoE4 plays a protective role against several main pathological events in AMD including inflammation, neovascularization and drusen formation; moreover, ApoE4 is associated with increased lipid transport across BM which is possibly due to its smaller size of ApoE/lipid complex compared to other isoforms and ApoE4 is relatively resistant to redox induced phase separation which is derived by mitochondrial fragmentation.

### Differential effects of ApoE isoforms on neurodegenerative disease of retina

ApoE4 and ApoE2 with abnormal function have been widely reported to be associated with the risk of developing several brain and retinal neurodegenerative diseases such as PD (Parkinson’s Disease) [53], AMD [23, 54], glaucoma [55] and DR [56]. While ApoE2 and ApoE4 have been widely reported by epidemiological studies as predisposing and protective factors respectively for developing retinal neurodegenerative disease [57, 58], these associations are in stark contrast to brain neurodegenerative disease, where ApoE2 and ApoE4 are shown to be protective and risk factors respectively [9]. There are however also conflicting results, with Adams et al (2012) reporting higher prevalence of early and late AMD in individuals carrying the E2 allele (OR: 1.32; 95%CI: 1.11, 1.58) and (OR: 1.77; 95%CI: 1.08, 2.99) respectively [9, 59, 60] in 2,287 AMD cases and 2,287 aged and sex matched controls. Surprisingly, there was no association between E2E2 and E2E4 genotypes with AMD, and only the E2E3 genotype showed a direct correlation. When results were stratified by smoking status these positive associations disappeared, interestingly the E4 allele did not show any correlation with AMD except in current smokers, which showed a protective effect against early AMD (OR: 0.41; 95%CI: 0.22, 0.77) [61]. Therefore, results of this study suggested smoking as a potential modifier of *APOE* polymorphism and AMD association and highlighted the significant effects of confounding factors on this association that should be taken into account in future studies.

### The role of ApoE isoforms on Bruch’s membrane (BM) permeability and the Extracellular Matrix (ECM)

The protective role of the ApoE4 isoform against AMD was first reported in 1998 in a study with 88 AMD cases and 901 healthy controls, which showed low prevalence of AMD particularly with soft drusen in E4 carriers, they suggested two possible biological mechanisms for the observed protective effect of ApoE4 against AMD which were related to Bruch's membrane (BM) [42]. BM is an elastin and collagen-rich extracellular matrix membrane that is located between the RPE and the retinal fenestrated choroidal capillaries that mediates transport between them. ApoE is required to maintain lipid clearance from the BM [62]. Ishida et al (2004) found that ApoE null mice showed lipid deposits in BM similar to those in AMD [62, 63]. ApoE4 may facilitate lipid clearance through BM with decreased permeability and increased thickness with aging, possibly due to deposition of lipid-rich inclusion bodies (Fig. 2). The contrasting roles between E4, E2 and E3 lie in the chemical structure. E3 does not have a cysteine at positions 112 and 158, therefore it is unable to form disulfide bridges with other peptide components or form dimers. Instead, it forms smaller lipid particles leading to better transport through BM [54, 57, 64, 65]. Likewise, the Extraceuular Matrix (ECM) compositions of both the retina and brain share similarities including the structure of both including proteoglycans, nonproteoglycan polysaccharides and other proteins such as fibronectin, lamnins and fibulins. Structurally, the ECM acts as a barrier to reduce the diffusion of soluble and membrane-associated molecules and cell migration[55, 66]. Recently it has been shown that APOE maintains arterial elasticity through the suppression of ECM matrix genes thereby conferring protection from cardiovascular disease[43].

### The role of ApoE isoforms on inflammation and neovascularization

Another proposed mechanism for the protective effect of the E4 isoform against AMD is through an anti-inflammatory effect via suppression of the expression of C-reactive protein (CRP), C-C motif Chemokine Ligand 2 (CCL2) and vascular endothelial growth factor (VEGF) [67]. The role of inflammation in the pathogenesis of AMD has been well established [42]; CCL2 is an inflammatory chemokine that has been shown to increase in AMD. With enhanced levels of CCL2 leading to increased macrophage proliferation and macrophage mediated VEGF and interleukin-6 (IL-6) expression [68]. VEGF has been observed in fibroblastic cells and choroidal neovascular membranes and implicated as a key player in aberrant choroidal neovascularization in AMD [42, 69]. Increased levels of both CCL2 and VEGF are associated with elevated oxidative stress within the retina, which mediates downstream pathological events in several retinal neurodegenerative diseases [70-72]. In contrast, IL6 induces an ocular inflammatory response such as triggering CRP production [73, 74]. The role of CRP in the pathogenesis of AMD has been well established and also showed to be a prominent component of drusen in AMD patients [75].With this in mind both E3 and E4 recombinant proteins reduced levels of CCL2 in RPE cells treated with lipopolysaccharide (LPS), however more reduction was observed in E4 compared to that of E3 group. A similar pattern of alteration was observed for VEGF upon treatment with ApoE isoforms, which supports the above mentioned mechanism for the protective effects of ApoE4 [76]. There are however conflicting results indicating an inflammatory role for ApoE in AMD that may contribute to mononuclear phagocyte (MP) accumulation and survival in the subretinal space in AMD patients. MPs are localised in the inner retina and their presence in the subretinal space is prevented by RPE immunosuppressive signals such as FAS ligand (FASL) [77]. In advanced stages AMD impaired FASL/FAS signaling leads to MP accumulation in subretinal space [77]. In AMD patients, subretinal MPs are accumulated on the RPE and release high levels of ApoE. Subsequent investigations in AMD mouse models have showed that *APOE* deletion decreased subretinal MP accumulation, photoreceptor degeneration and choroidal neovascularization (CNV) [77]. Moreover, it has been shown that ApoE increases the expression of IL-6 in a TLR2-CD14 dependent manner, and IL-6 decreases the expression of FASL from RPE and this prevents MP elimination from subretinal space. Moreover, ApoE increased the expression of CCL2 which is shown to be involved in accumulation of inflammatory MPs in the subretinal space [77, 78]. Furthermore, macrophages of mice carrying the E2 isoform released higher levels of ApoE compared to other isoforms [79]. Accordingly, both reduced clearance and increased production of ApoE leading to higher levels of IL-6, CCL2 and IL-1β and innate immunity receptor cluster (IIRC) activation. This was followed by subretinal inflammation, photoreceptor degeneration and CNV formation [79, 80]. The E4 isoforms is associated with decreased levels of ApoE, IL-6 and CCL2 and consequently reduced subretinal inflammation, photoreceptor degeneration and CNV formation [77, 79, 81]. In these studies, AMD mouse models showed subretinal inflammation and associated photoreceptor degeneration as two established hallmarks of AMD, but did not fully mimic the AMD pathological changes including drusen formation and RPE atrophy. The effects of APOE on VEGF and inflammatory cytokines was also confirmed in another study that explored upstream targets of APOE [82]. Considering the role of mitogen-activated protein kinase (MAPK) signaling pathways including ERK1/2, JNK1/2/3, p38 MAPK and ERK5 in modulating the expression of VEGF, IL-1b, IL-6 and TNF-a in neovascular AMD (nvAMD) development [83-85]. The effects of APOE isoforms on MAPK signaling in human RPE cells and laser-induced nvAMD mouse models expressing human APOE2 isoform were evaluated. Results demonstrated that APOE2 activates MAPKs signalling (p-ERK, p-JNK and p-p38) and induces the up-regulation of downstream target genes including VEGF, PDGF-BB and b-FGF growth factors and pro-inflammatory molecules such as TNF-a, IL-1b and IL-6, leading to retinal inflammation and nvAMD development [82]. These findings highlight the protective role that the APOE e4 allele plays in AD and the pro inflammatory role in AMD. This further highlights the importance of understanding the role of the E4 allele in retinal neurodegenerative changes in ophthalmic disorders.

### ApoE isoform dependent drusen formation

The RPE is a monolayer of postmitotic polarized cells located between the neuroretina and the choroid and provides biochemical support to photoreceptor cells [86]. RPE dysfunction is an early event in AMD which is associated with the formation of lipid-protein aggregates called drusen, which deposit in the sub-RPE space [87]. Drusen size, localisation and morphology are known to be predictors of AMD progression [86]. Cholesterol and the ApoE protein are the two primary components of drusen in AMD eyes, however the effects of *APOE* polymorphism on drusen formation are not well defined [45, 88]. La Cunza et al (2021) showed in adult primary RPE monolayers, that ApoE3 and ApoE4, but not ApoE2 effectively inhibited the bis-retinoid induced cholesterol increase [89]. In ApoE2 carriers elevated cholesterol levels resulted in acid sphingomyelinase (ASMase) activation, that hydrolyzes sphingomyelin to ceramide [89]. Elevated level of ceramide impair autophagosome biogenesis and trafficking, endosome biogenesis, and lysosomal localization through tubulin acetylation in RPE cell cultures expressing cherry tagged APOE [89]. Similar findings were observed in Abca4^-/-^ mice which have higher rates of vitamin A dimerization and lipofuscin accumulation. ApoE2 isoform but not ApoE3 and ApoE4, acetylated microtubules impaired complement-regulatory mechanisms leading to complement mediated cell membrane defects which resulted in increased intracellular calcium level and calcium mediated mitochondrial fragmentation [89]. In addition, results of this study highlighted the underlying mechanism of drusen formation which was triggered by ApoE2 phase separation. Liquid-liquid phase separation of proteins is accompanied by intrinsically disordered regions (IDRs) that enabled their aggregation via low-affinity interactions, following mitochondrial fragmentation. ApoE2 condensates were observed, and small but significant ApoE3 condensates also were detected which may nucleate the drusen. However, no ApoE4 condensates were observed, which indicated that this isoform may be resistant to phase separation (Fig. 2). Finally, it has been concluded that mitochondrial damage drives redox-mediated phase separation in ApoE2 and ApoE3 but not ApoE4 possibly due to lack of cysteine residues in its structure [89-93].

### ApoE genotype role in precision medicine

*APOE* polymorphisms can influence the treatment outcomes in AMD patients. A study that included 192 nvAMD (Neovascular age related macular degeneration) patients who received anti-VEGF treatment found that the APOE ε4 allele was significantly associated with significant visual improvement compared to APOE alleles at 3,6 and 12 month after treatment initiation [94]. This effect was also confirmed in another study that examined the effect of *APOE* polymorphism on response to ranibizumab (a humanized anti- VEGF antibody) in 109 exudative AMD patients [95]. Results of this study showed improvement of visual activity in AMD patients carrying E4 allele and indicated an association between E4 allele and visual improvement in AMD patients using ranibizumab. While no association was observed for the E2 allele [95]. However, the sample size of this study was relatively small and there were no patients classified with the E4/E4 genotype with only one patient with the E2/E2 allele. Taken together, these results highlight the potential impact of *APOE* genotypes on treatment response in AMD.

## ApoE in AD

In the brain, ApoE is mainly synthesized by non-neuronal cells including astrocytes and to some extent by microglia. In excitotoxic injury low levels of ApoE are also expressed by neurons [96-99]. ApoE is the main component of HDL-like particles within the brain with a primary role in cholesterol and phospholipid transfer between cells, however it is also involved in glucose metabolism and insulin signaling, immunomodulation, transcription and proteostasis regulation [38, 100]. Interestingly ApoE has profound effects on regulating Aβ metabolism, tau pathology, mitochondrial function, inflammation and other pathways that play a central role in AD pathogenesis [7, 9, 38, 40, 99, 101, 102] therefore it is suggested that ApoE isoforms may differentially influence AD initiation and progression pathways. In the brain, cellular cholesterol and phospholipid efflux from cells is mediated by ATP-binding cassette transporter (ABCA1), with ApoE polymorphisms also showed to be associated with AD [103].

## Effects of ApoE polymorphism in AD

The ApoE4 isoform is now considered as the strongest genetic risk factor for late onset AD while ApoE2 is suggested to have some protective effects [9, 38].

**Figure 3.**
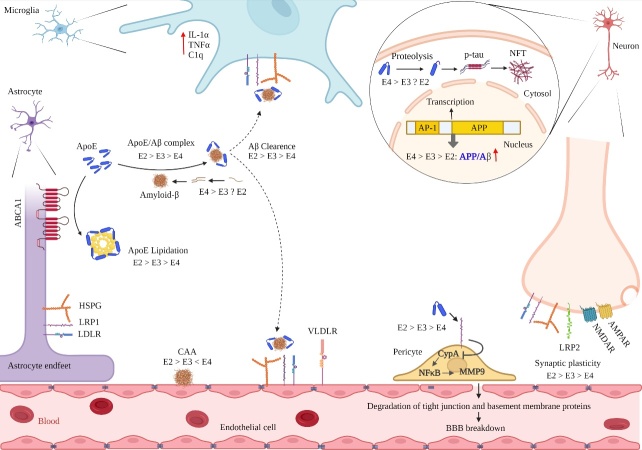
**Differential effects of ApoE isoforms on AD pathological events**. ApoE4 considered as a risk factor for developing AD and is correlated with several pathological events in this disease; ApoE4 is associated with decreased synaptic plasticity, increased Aβ production and aggregation, decreased Aβ clearance, increased NFT formation, increased levels of ApoE fragments, increased inflammation, and level of MMP9 which drivers of BBB breakdown.

Isoform dependent effects of ApoE on Aβ metabolism is suggested as a major reason for the association of ApoE polymorphism with AD [104]. Co-deposition of ApoE with Aβ in senile plaques was observed about three decades ago and indicated a direct correlation between ApoE and Aβ in AD [105-107]. There are conflicting reports about the effects of ApoE on Aβ aggregation. While some studies reported ApoE targeting as a therapeutic approach to reduce Aβ aggregates [102, 106, 108, 109], ApoE deletion results in improved neuronal responses, reduced neuritic plaques, reactive glia and synaptic loss in a transgenic mouse model of AD [110].. Residues 144-148 in the N-terminal region of ApoE and residues 13-17 in Aβ are involved in ApoE/Aβ complex formation; interestingly, residues 244-272 in the C-terminal region of ApoE that are within the lipid binding site are also required for its ApoE/Aβ complex formation. Moreover, heparan sulfate proteoglycan (HSPG), is the major cell surface receptor for both ApoE and Aβ [111-113]. These reports indicate that Aβ can binds to both lipid binding and receptor-binding sites of ApoE in vivo, therefore its presence can interfere with APOE’s role as a lipid carrier [32, 113, 114]. Interestingly increased ApoE lipidation in ABCA1 transgenic mice leads to decreased Aβ deposition and ABCA1 deletion resulted in increased brain Aβ and amyloid load[40, 115].ABCA1 may facilitate ApoE lipidation and inhibit ApoE/Aβ complex formation (Fig. 3).

The E4 isoform is associated with increased senile plaque formation in AD and is considered as a risk factor for developing AD, whereas the E2 isoform is associated with lower levels of senile plaques and decreased risk of developing AD [79]. The mechanisms underlying these isoform dependent effects are not fully understood, however ApoE isoform interactions with Aβ and their effects on Aβ aggregation and clearance are suggested to be major mechanisms that underlie this association. ApoE isoforms have different Aβ binding efficiency; E2 and E3 bind Aβ more efficiently than E4 which effects their abilities in Aβ aggregation and clearance (E2 > E3 > E4) leading to increased Aβ aggregation in E4 carriers [9, 112, 116-118]. Moreover, lower levels of ApoE in E4 carriers may exacerbate this condition and contribute to Aβ cytotoxicity in humans [119]. Cramer et al (2012) showed that increasing the expression of ApoE in a mouse model of AD by a retinoid X receptor (RXR) agonist rapidly enhanced the clearance of Aβ from the CNS [120]. Additionally, E4 isoform is more susceptible to proteolysis than other isoforms and E4 fragmentation has been suggested as an early event in AD pathogenesis. Studies reported that ApoE4 fragments but not E3 fragments accelerate Aβ aggregation and suggest that these fragments but not E4 itself as a risk factor for AD pathogenesis [121-124].

There are several other proposed mechanisms explaining increased Aβ aggregation and decreased clearance in E4 carriers [125]; Aβ aggregation is a nucleation dependent process, starting with a slow nucleation when free monomers are added onto a nucleus and forming small aggregates and extending rapidly in amyloid growth stage [125]. ApoE has been shown to target Aβ fibril nucleation and elongation and impair amyloid maturation; however, this inhibitory effect is lower in ApoE4 compared to the other isoforms. ApoE4 accelerates early seeding of amyloids, which ultimately leads to enhanced amyloid deposition and down-stream pathological events [104, 126, 127]. Besides, ApoE polymorphism also affects the response to AD treatments. Incidence of amyloid-related imaging abnormalities - edema/effusion (ARIA-E) showed to be higher in ApoE4 carriers compared to non-carriers following treatment with Lecanemab, as an anti-Aβ monoclonal antibody approved by the US Food and Drug Administration (FDA) in Janeury 2023 [128, 129]. Therefore, ApoE polymorphism should be considered in clinical trials to better evaluate threaputic and side effects of AD potential drugs in different ApoE carriers.

BBB-associated pericytes have been implicated to mediate uptake of Aβ through a LRP1/ApoE isoform-specific mechanism, which is lower in E4 carriers compared to E3 carriers [100]. Additionally, the weaker inhibitory effect of the E4 isoform against Aβ fibril nucleation and elongation leads to amyloid aggregation and following amyloid-induced toxicity on blood vessel pericytes [130, 131]. ApoE also regulates MMP-9 function in an isoform dependent manner. MMP-9 is a Zn^2+^ dependent endopeptidase that increases lipoprotein receptor shedding and leads to decreased Aβ clearance across the BBB, blocking this enzyme results in increased Aβ transit across the BBB. The E4 isoform weakly inhibits MMP-9 function compared to other isoforms causing lower Aβ clearance across BBB [132, 133].

As previously discussed the E4 isoform is associated with lower level of HDL and elevated levels of HDL and is associated with decreased risk of developing AD [39] . HDL-mimetic peptide 4F has been shown to increase ApoE secretion and lipidation in primary human and mouse astrocytes and reduce Aβ aggregation [37, 39] Therefore lower levels of HDL in E4 carriers have been observed, resulting in decreased ApoE secretion and lipidation [40, 134]. Alternatively, ApoE promotes Aβ degradation through decreasing cellular cholesterol levels [104, 135], therefore elevated levels of cholesterol in E4 carriers may reduce intracellular trafficking of Aβ to lysosomes and suppress its degradation. Apart from the role of ApoE in aggregation and clearance of Aβ, the role of ApoE in stimulating neuronal Aβ secretion also has been reported. A study using embryonic stem cells-cell-derived human neurons showed that ApoE binding to its receptors led to activation of dual leucine-zipper kinase (DLK), a serine/threonine protein kinase, which stimulates MKK7 and ERK1/2 MAP kinases, subsequently resulting in increased APP transcription and elevated Aβ levels. The results of this study also showed isoform dependent effects of ApoE on APP transcription and Aβ production, where ApoE4 was associated with higher levels of Aβ production followed by E3 and E2 (ApoE4 > ApoE3 > ApoE2) [136]. However studies on mice models of AD yielded contradictory results, indicating no association between ApoE isoforms and APP transcription [118, 137]. These differences highlight the importance of human studies as different ApoE isoforms are only expressed in humans and transgenic animals expressing human genes, may not reliably replicate the disease condition.

### Effects of ApoE isoforms on Tau protein

Abnormal deposition of phosphorylated tau (p-tau) protein is a hallmark of several neurodegenerative disease known as tauopathies [138]. There are several conflicting results about the association of ApoE with tau protein phosphorylation and aggregation [139, 140]. Isoform dependent effects of ApoE on tau aggregation and neurofibrillary tangle formation (NFT) have been reported; E2 and E4 isoforms are considered as either protective or risk factors respectively for tau aggregation and higher p-tau load in AD patients [141, 142]. However, this association is observed only in presence of Aβ pathology, and in primary tauopathies without amyloid deposition such as observed in progressive supranuclear palsy (PSP). There are also conflicting results indicating association of the E4 isoform with enhanced tau aggregation independent from Aβ burden [143]. An association of carboxyl-terminal-truncated forms of ApoE4 with NFT-like inclusions in neurons has been reported in brain of AD patients [144]. Increased levels of cholesterol and low levels of HDL in E4 carriers also may contribute to enhanced levels of p-tau and its aggregation [145, 146]. Preclinical AD and longitudinal hippocampus changes have been reported in E4 and non carriers in the early clinical years (7-13 yrs pre-AD). The E4 carriers showed eleveated hippocampal connectivity at rest with p-tau181 and subsequent hippocampus encoding activity [147]. Similarily Young et al (2023) looked at APOE effects on regional tau in preclinical AD and found that APOE influences early regional tau burden beyond cross sectional amyloid burden [148].

### Effects of ApoE isoforms on neuroinflammation

Neuroinflammation is major pathological feature of AD, which is mediated by microglia and astrocytes that release anti/pro-inflammatory cytokines [149, 150]. Exposure to Aβ activates microglial activation that may damage neuronal cells [151]. Moreover activated microglia release IL-1ɑ, TNFɑ and C1q and activates astrocytes [152]. ApoE has been shown to inhibit inflammation and reduce classical complement cascade (CCC) activation via binding to the activated CCC-initiating C1q protein [153]. A mutation in a microglial receptor, termed triggering receptor expressed on myeloid cells 2 (TREM2), which is a receptor for ApoE, has been shown to be associated with increased risk of developing AD [154, 155]. Up-regulation of TREM2 receptor has been observed in AD and deletion of this receptor impaired microglia migration, with increased amyloid seeding in the early stage of amyloidogenesis and reduced plaque-associated ApoE. This suggested a key role of TREM2 in microglia mediated Aβ clearance. It has been shown that in early Aβ deposition expression of TREM2 is protective, while in later stages could be detrimental [156-159]. Microglia and astrocytes are two main sources of ApoE in the brain. Studies have shown the anti-inflammatory effects of ApoE in preventing microglial activation in response to lipopolysaccharide (LPS) and APP in an isoform dependent manner [160, 161]. There are also reports indicating pro-inflammatory features of ApoE in response to LPS leading to increased Aβ deposition in APP mice [162]. ApoE deficient mice at the age of 3, and 9 months showed different levels of p-tau, brain atrophy and neuroinflammation in ApoE carriers with significant elevation in E4. ApoE deficient mice however did not show these changes; moreover, treatment with LPS showed a higher innate immune reactivity and TNFα secretion in E4 carriers. Similarly, an association of E4 isoform with microglia activation and tau pathology independent of Aβ pathology was also observed in the dorsolateral frontal cortex of AD patients. These findings collectively suggest isoform dependent effects of ApoE on neuroinflammation, tau aggregation and p-tau mediated neurodegeneration independent of Aβ pathology, where ApoE deficiency may be protective [163, 164]. An Isoform dependent effect of ApoE on microglia activation was also observed in APP transgenic mice, whereas E4 was associated with higher Aβ deposition, reactive microglia, and dystrophic astrocytes. Interestingly increased microglial reactivity was greater in E3 compared to E2 (E4˃ E2˃ E3) [165]. Dorey et al highlighted that lipidated ApoE4 but not free ApoE4 promoted Aβ induced inflammation, whereas lipidated ApoE3 and both lipidated and free ApoE2 inhibited Aβ induced inflammation. They showed that ApoE2 was found to activate vitamin D receptor (VDR) in Aβ42-treated astrocytes and that 1α,25-dihydroxyvitamin D3 treatment resulted in decreased Aβ-induced inflammation in ApoE4 expressing astrocytes. These results suggest a key role of VDR signaling in the differential effects of ApoE isoforms on inflammation [166]. Variations might be due to their different effects on Aβ aggregate formation following Aβ induced neuroinflammation. Differences might also be independent from Aβ aggregates and more associated with changes in levels of cholesterol and HDL in E4 carries that play a modulatory role in neuroinflammation [167, 168].

### Effects of ApoE isoforms on cerebrovascular system

The cerebrovascular system functions to provide nutrients and oxygen to the brain. It is essential for proper brain function with impaired cerebrovascular structure and function being associated with several neurodegenerative diseases. ApoE plays a key role in BBB (Blood Brain Barrier) integrity and ApoE deletion leads to BBB impairment. BBB dysregulation has been suggested as an early event in the hippocampus in AD [169, 170]. The BBB is formed by three cells types including brain endothelial cells (BECs), pericytes and astrocytes [171]. It has been shown that in mice that ApoE supports BBB integrity through controlling the expression of cyclophilin A (CypA) in pericytes via LRP1. CypA is a proinflammatory cytokine that activates the CD147 receptor and downstream nuclear factor-kB- MMP9 (CypA-NF-kB-MMP9) pathway in pericytes however ApoE4 but not ApoE2, ApoE3 and mice ApoE, were inefficient in controlling CypA expression in pericytes leading to higher levels of MMP9 and consequently BBB breakdown [172, 173]. MMPs affect ECM, tight junction components, growth factors and their precursors, cell surface receptors and cell adhesion molecules. Although uncontrolled expression of MMPs can result in tissue injury and destruction such as BBB dysfunction, its expression is required for neuronal plasticity and vascular remodeling [174, 175]. Elevated MMP9 expression in ApoE4 carrier induces BBB breakdown through enzymatic degradation of the BBB tight junction and basement membrane proteins. BBB leakage leads to blood-derived neurotoxic proteins uptake by neurons, perivascular deposition of erythrocyte-derived hemosiderin and microvascular and consequently decreased cerebral blood flow [172, 176, 177]. Higher pericytes degeneration and less BBB integrity have been also observed in AD patients carrying E4 isoform compared with E3 and E2 carriers [170, 178].

APOE exists in two distinct pools in the body (Peripheral and CNS). Whilst the brain and peripheral pools of APOE are distinct, APOE shares similar systemic effects in these two distinct locations namely regulating lipoprotein metabolism and cellular differentiation [179]. A similar effect is seen in the role of APOE and tau. In the CNS APOE4 stimulates phosphorylation of tau however in the periphery APOE may also act on tau. Badia et al (2013) showed that APOE4 individuals havd more more phosphorylated tau in circulating peripheral lympho-cytes than their APOE3 counterparts [180].

Cerebral amyloid angiopathy (CAA) is a condition caused by the accumulation of cerebral Aβ in the walls of the small and medium arteries in the brain and is associated with brain hemorrhages particularly microhemorrhages [181, 182]. CAA is common concurrence in AD and is affected by ApoE in an isoform dependent fashion. In human stem cell-based three-dimensional models of BBB, higher Aβ deposition in BBB was observed in ApoE4 carriers compared to ApoE3 carriers and the presence of pericyte-like mural cells are required for Aβ deposition in the BBB as up-regulation of ApoE and calcineurin-nuclear factor of activated T cells. NFAT (Nuclear Factor of Activated T Cells) signaling in these cells was shown to be critical for ApoE4 induced CAA [171]. Interestingly targeting ApoE that is co-deposited with Aβ in CAA in a mouse expressing human ApoE4 not only efficiently reduced parenchymal Aβ plaques and CAA, but also ameliorated reactive microglial, astrocytic and expression of proinflammatory genes. Treating with anti-ApoE did not caused brain hemorrhages that are components of amyloid-related imaging abnormalities (ARIA), a common side effect of anti-Aβ antibodies in blood vessels with CAA [183, 184]. Surprisingly ApoE2 is associated with increased severity of CAA compared to ApoE3, both E2 and E4 are associated with CAA, but E2 carriers are at a higher risk of ruptured blood vessel and resultant hemorrhages [185]. On the other hand, E4 carriers showed more microbleeds than hemorrhages. Interestingly these isoforms affect vessels with different sizes, where E4 but not E2 is implicated in capillary amyloid angiopathy. The reasons for this size dependent action are currently unclear, however different ApoE receptors or their different size or their ability in forming the dimers might be involved [37, 57, 186].

### Effects of ApoE isoforms on synaptic function and VEGF signaling

Synaptic dysfunction is an early pathological event in AD that has been suggested to occur before significant neurodegeneration and memory dysfunction [187-189]. Detrimental effects of Aβ deposits on synaptic function have been well established therefore ApoE isoform differential affects synaptic function through an Aβ dependent manner however there are also some Aβ independent mechanisms through which ApoE may play a role [118, 190]. ApoE is considered as a protective factor in neuronal injury through providing sufficient cholesterols and lipids for neurons and also stimulating neurite outgrowth, which is promoted by ApoE3 more effectively than ApoE4, or even inhibited by ApoE4 [190, 191]. ApoE4 is associated with lower dendritic spine density and length compared to ApoE3 carriers and is associated with cognitive impairment even early in life [192-194]. ApoE4 is associated with reduced levels of two ApoE receptors including LRP1, which is involved in neurite outgrowth, and Apolipoprotein E receptor 2 (ApoER2) which is also a receptor for Reelin. A glycoprotein involved in synaptic function in adulthood [195-197].

Vascular endothelial growth factor (VEGF) is involved in neurogenesis, neuronal plasticity, and repair [198-201]. Reduced levels of VEGF have been reported in AD patients while decreased levels of VEGF may contribute to neurodegenerative diseases such as Parkinson’s and multiple sclerosis (MS) through affecting neuronal plasticity and survival [196, 202] [203]. Increased levels of VEGF may induce BBB breakdown and vessel leakage, therefore both administration of VEGF and anti-VEGF may be an effective treatment in neurodegenerative diseases depending on the main pathological events of the disease [203]. VEGF is beneficial for neurogenesis, neuronal plasticity and neuronal survival. alternatively, anti-VEGF can ameliorate BBB breakdown or excessive angiogenesis [204]. Interaction of ApoE polymorphism with VEGF genes has been shown to affect cognitive impairment in AD patients as increased levels of Neuropilin 1 (NRP1), a member of VEGF family in AD patients has been shown to be associated with improved cognition in ApoE4 non-carriers. [205]. In AD mouse models, significantly lower levels of hippocampal VEGF and its receptor (VEGFR-2) have been reported in ApoE4 carriers compared to ApoE3 carriers, resulting in higher Aβ deposition, p-tau aggregates, synaptic and cognitive impairment, which were then resolved by overexpressing VEGF[196, 206].

### Effects of ApoE isoforms on glucose metabolism

Reduced glucose metabolism due to insulin resistance is a key pathogenic mechanism of Type 2 Diabetes (NIDMM) [207]. Interestingly this pathological mechanism also plays an important role in AD [208, 209]. The brain is one of the most energy demanding organ within the body and glucose is the major source of energy for neurons [210]. Impaired cerebral glucose metabolism is an early pathological event in AD that occurs even decades before cognitive impairment and histopathological changes and ApoE4 is associated with higher level of impaired glucose metabolism [211-213]. The mechanisms underlying differential effects of ApoE isoforms on glucose metabolism are not still clear, however there are several reports providing valuable clues for this association [118, 214, 215]. Interestingly ApoE4 but not Aβ aggregates were shown to be correlated with lower glucose metabolism and insulin signaling in cognitively normal older people, in addition in transgenic mice expressing human ApoE, ApoE2 increased glucose metabolism independent from soluble Aβ_40_ or Aβ_42_ levels. [216, 217]. ApoE also affects glucose utilization in astrocytes via an isoform dependent manner, with glycolysis higher in ApoE2 compared to other isoforms. While ApoE4 is associated with the highest level of lactate synthesis from glucose, ApoE4 shifts glucose flow towards the pentose phosphate pathway (PPP) and tricarboxylic acid cycle (TCA cycle). This results in higher gluconeogenesis, lipid and de novo nucleotide biosynthesis [218]. Furthermore ApoE4 is associated with decreased energy expenditure and decreased glucose oxidation via increasing flux through aerobic glycolysis (i.e. the Warburg effect) [219, 220]. In line with these, in ApoE4 transgenic mice the expression of glucose transporter 3 (GLUT3), which is the main GLUT isoform in the neuron populations was found to be decreased compared to ApoE3 carriers. The expression of GLUT1, which is involved in glucose transport across the BBB, was not changed despite significant reduction of glucose transport, indicating a role of posttranslational modifications underlying dysfunctional GLUT1. However overexpressing GLUT1 in the neurons of adult flies results in reduced Aβ related toxicity [210, 214, 221]. In addition, defective insulin/insulin-like growth factor (Igf) and peroxisome proliferator-activated receptor γ (PPARγ) and PPARγ coactivator 1 (PGC1α) signaling, was observed in ApoE4 carriers and PGC-1ɑ overexpression in ApoE4 carriers reduced these changes through increased mitochondrial biogenesis, respiratory capacity and oxidative phosphorylation [214, 215].

**Figure 4.**
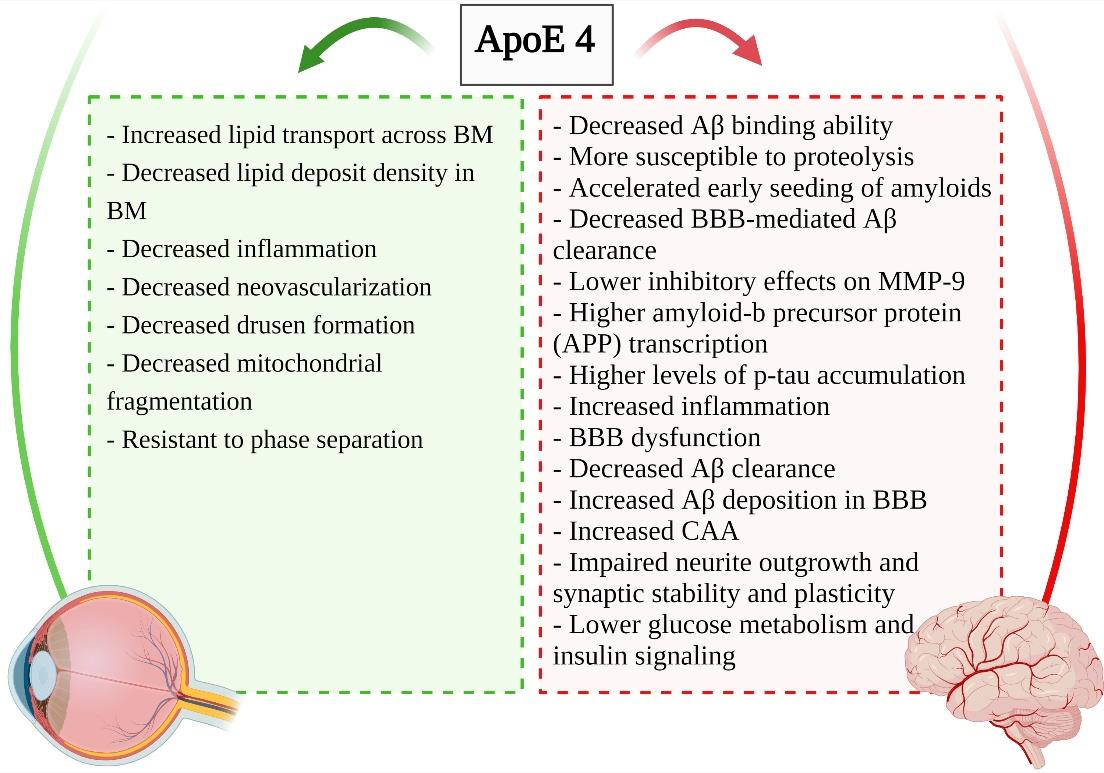
Schematic presentation of the differential effects of ApoE4 in AMD and AD.

## Conclusion

A growing number of studies have implicated a link between AD and retinal neurodegenerative disease that share several pathological events and risk factors. ApoE4 is the strongest genetic risk factor for AD whereas ApoE2 is considered as a protective factor. However, in retinal neurodegenerative diseases paradoxically ApoE4 has been suggested as a protective factor and ApoE2 is the risk factor [77, 79]. Although there are many reports on the effects of ApoE isoforms on AD initiation and progression, data about their role in retinal neurodegenerative diseases are limited and further studies are needed to reveal their differential effects on pathogenesis of these diseases. Currently several Aβ dependent and independent mechanisms have been suggested for differential effects of ApoE isoforms in the pathogenesis of the aforementioned diseases (Fig. 4). However further research is needed to unravel their differents role in these diseases. In addition, effects of gender, environmental factors, ethnicity, and lifestyle such as diet, exercise, smoking status, need to be investigated in both epidemiological and experimental studies as the important modifiers of ApoE effects.
